# C-Type Lectin Receptors in Antiviral Immunity and Viral Escape

**DOI:** 10.3389/fimmu.2018.00590

**Published:** 2018-03-26

**Authors:** Marta Bermejo-Jambrina, Julia Eder, Leanne C. Helgers, Nina Hertoghs, Bernadien M. Nijmeijer, Melissa Stunnenberg, Teunis B. H. Geijtenbeek

**Affiliations:** ^1^Department of Experimental Immunology, Amsterdam Infection and Immunity Institute, Academic Medical Center, University of Amsterdam, Amsterdam, Netherlands; ^2^Division of Hygiene and Medical Microbiology, Medical University of Innsbruck, Innsbruck, Austria

**Keywords:** C-type lectin receptors, antiviral immunity, antigen presentation, type I IFN, complement opsonized HIV-1

## Abstract

C-type lectin receptors (CLRs) are important pattern recognition receptors involved in recognition and induction of adaptive immunity to pathogens. Certain CLRs play an important role in viral infections as they efficiently interact with viruses. However, it has become clear that deadly viruses subvert the function of CLRs to escape antiviral immunity and promote infection. In particular, viruses target CLRs to suppress or modulate type I interferons that play a central role in the innate and adaptive defense against viruses. In this review, we discuss the function of CLRs in binding to enveloped viruses like HIV-1 and Dengue virus, and how uptake and signaling cascades have decisive effects on the outcome of infection.

## Introduction

**Figure 1:**
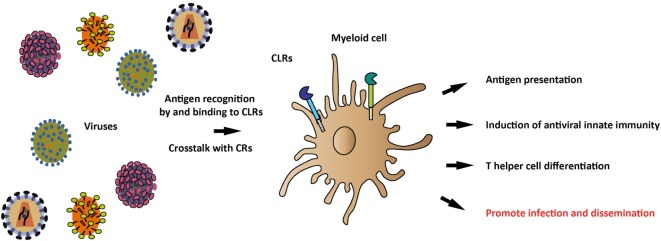
Recognition of viral antigens by C-type lectin receptors (CLRs) and induction of antiviral immune responses. Various CLRs on antigen-presenting myeloid cells recognize a plethora of viruses through their carbohydrate-recognition domains (CRDs) and subsequently induce a tailored immune response, depending on the specific CLR and viral antigens. Viral antigens that trigger the CLR can modulate myeloid cell functions, thereby affecting antigen presentation, antiviral innate immune responses, and T helper differentiation. Once viral antigens are recognized by CLRs, crosstalk between CLRs and complement receptors (CRs) can occur, thereby further shaping the antiviral immune response. Additionally, CLRs play a role in viral recognition, internalization, and dissemination.

Mucosa and skin are targets for invading viruses and are therefore important sites where adaptive immunity is initiated. Dendritic cells (DCs) and macrophages guard these tissues and detect the invading pathogens by pattern recognition receptors (PRRs) and lead to initiation of immunity and elimination of the pathogens (Figure 1). DCs are professional antigen presenting cells (APCs) that capture pathogens for degradation and antigen presentation, whereas macrophages are a first line of defense that destroy pathogens *via* degradation but are also able to activate memory T cells (1–3). PRRs are crucial for these functions of DCs and macrophages, as PRRs recognize conserved molecular structures to distinguish between the different types of pathogens, called pathogen-associated molecular patterns (PAMPs) (4). Distinct classes of PRRs recognize a wide range of PAMPs and induce different transcriptional programs leading to tailored immune responses. Furthermore, pathogens will often trigger several PRRs, leading to crosstalk between these receptors, which provide immune cells with another important level of control to tailor the adaptive immune response to the pathogen. Several classes of PRRs exist, such as Toll-like receptors (TLRs), Rig-I-like receptors (RLRs), and C-type lectin receptors (CLRs). Here, we will focus on CLRs that recognize carbohydrate structures, which are able to independently induce immunity or provide powerful signals *via* crosstalk to modulate responses that are triggered by other PRRs (5). As the immune system is in a never-ending arms race with viruses, many viruses have devised strategies to evade recognition and antiviral immune responses to successfully infect the host. In this review, we describe the complex role of CLRs in the immune processes that are essential in the defense against viruses. In addition, we discuss how certain viruses target specific CLRs to suppress or avoid antiviral immunity.

## CLRs Interact with Viruses, Leading to Virus Degradation or Transmission

### Virus Recognition

**Figure 2:**
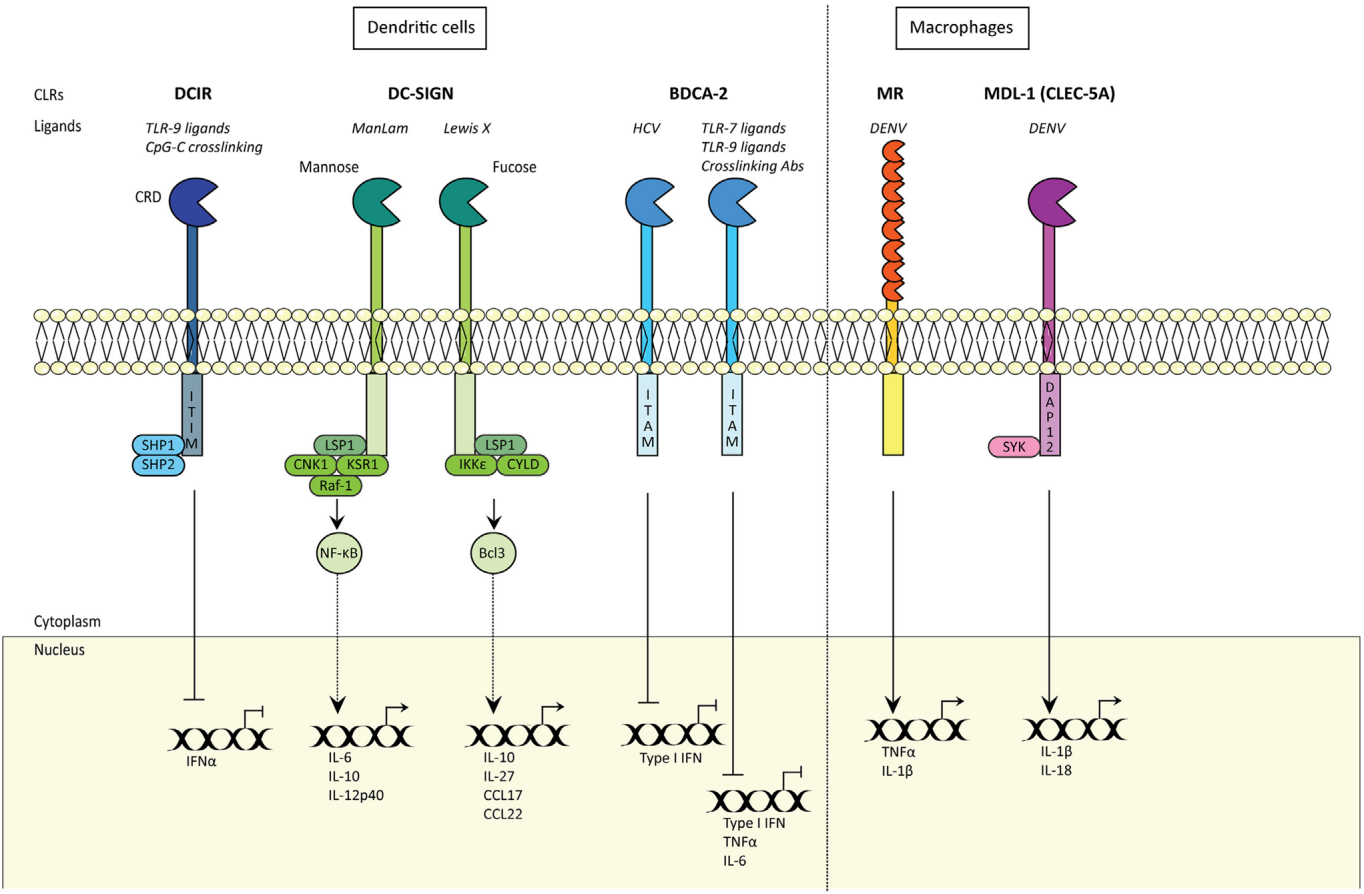
C-type lectin receptors (CLRs) shape innate and adaptive immune responses. CLRs induce innate and adaptive immune responses. Certain CLRs contain ITIM domains and signal *via* SHP1 and SHP2 phosphatases, whereas other CLRs signal *via* their ITAM motif. DC-SIGN signaling is carbohydrate specific (CRD) and either signals *via* Raf-1 signalosome or IKKε and de-ubiquitinase CYLD, with distinct outcomes. MDL1 signals *via* DAP12 and Syk. CRD; carbohydrate recognition domain; DCIR, DC immunoreceptor; TLR9, Toll-like receptor 9; ITIM, immunoreceptor tyrosine-based inhibitory motif; SHP1, SH2-domain-containing protein tyrosine phosphatase 1; DC-SIGN, DC-specific ICAM-grabbing non-integrin; IFNα, interferon alpha; LSP1, lymphocyte-specific protein 1; CNK1, Connector Enhancer of KSR1; KSR1, kinase suppressor of Raf-1; IKKε, IκB kinase subunit-ε; IL6, interleukin 6; CCL17, Chemokine (C-C motif) ligand 17; NF-κB, nuclear factor kappa beta; Bcl-3, NF-κB family member Bcl-3; KSR1 and CNK1 are adaptor proteins; BDCA-2, blood DC antigen 2; HCV, hepatitis C virus; ITAM, immunoreceptor tyrosine-based activation motif; TNFα, tumor necrosis factor alpha; Type I IFN, type I interferon; MR, mannose receptor; DENV, Dengue virus; MDL-1, myeloid DAP12-associating lectin-1.

Innate immune cells like monocytes, macrophages, DCs, and Langerhans cells (LCs) express CLRs that act as PRRs. Most of these CLRs bind carbohydrate moieties in a calcium-dependent manner using conserved carbohydrate recognition domains (CRDs) (Figure 2). CLRs are important for recognition and capture of pathogens as these PRRs have a high affinity for their ligands, which results in internalization of the pathogens. Internalization often leads to degradation *via* lysosomes, which has been shown for the DC-specific ICAM-3 grabbing non-integrin (DC-SIGN; CD209) and DEC-205 (6, 7), or the binding induces degradation *via* autophagy as shown recently for langerin (8). Therefore, the outcome of CLR recognition depends on the specific CLR and the cell type on which it is expressed.

### Lysosomal Degradation and Virus Transmission

After binding of pathogens by CLRs, the routing (intracellular transport) of antigen has various outcomes depending on the CLR and the immune cell. The Mannose Receptor (MR) is expressed by macrophages and DCs, and is involved in antigen processing and presentation. MR recognizes mannose, *N*-acetylglucosamine, and fucose that are often found on the surfaces of viruses, bacteria, and parasites (9). Upon binding, the pathogen is internalized and targeted to lysosomes for degradation. Subsequently, MR recycles back to the cell surface, for the next round of internalization, resulting in high amounts of internalized pathogens (10). However, several studies have shown that viruses such as HIV-1 and Dengue virus (DENV) target MR to evade degradation (11). Besides MR, the CLR DC-SIGN also plays an important role in virus binding and internalization. DC-SIGN recognizes mannose and fucose structures (12–14). HIV-1 internalization within DCs is dependent on the association between gp120 and DC-SIGN and this interaction can deliver HIV-1 to lysosomes where they are degraded (6). However, strikingly, a major part of DC-associated HIV-1 evades the degradation pathway by trafficking to a tetraspanin (CD81)-enriched protective environment from where infectious particles are specifically released to T lymphocytes upon DC–T cell contact (15). Thus, a virus that is taken up by DCs can enter two pathways: either routed to the endocytic pathway, resulting in viral degradation and antigen presentation or diverted to a transmission pathway and thereby avoids degradation. It is unknown how these pathways are related and which factors determine the fate of the virus. Langerin is a CLR expressed exclusively on LCs and is important for antigen capture and internalization, which induces Birbeck granules (BG) formation and routing of antigen into organelles (16). Langerin has a role in antiviral protection as immature LCs capture HIV-1 *via* langerin, leading to TRIM5α-mediated autophagic degradation of HIV-1, which prevents LC infection (8, 17). Thus, CLRs are important in the final fate of the virus, which can be either routing for degradation or dissemination.

### Antigen Presentation

Next to routing of antigen in lysosomal pathways for degradation, antigen presentation on major histocompatibility complex (MHC) molecules is an important anti-viral immune mechanism. Many antigens taken up by various CLRs such as DC-SIGN, DEC-205, DCIR, or dectin-1 are routed into MHC class II compartments, where the antigens are loaded for presentation to CD4 + T cells (18–21). These MHC class II molecules are released from late endocytic compartments and accumulate at the cell surface (22). The expression of most CLRs is generally downregulated upon cell maturation (23). Mature DCs have a reduced capacity to take up antigen, which is reflected by lower levels of CLRs, but are more efficient in stimulating T cells through stabilizing MHC class I and class II at the cell surface (24). An exception represents DEC-205, as the receptor is upregulated in mature plasmacytoid DCs (pDCs) (25). Importantly, antigen presentation by DEC-205 is not affected by this as endocytosis of an antigen targeted to DEC-205 *via* an antibody still leads to antigen presentation and CD4 + T cell induction in immature as well as activated pDCs (25). Furthermore, CLR Siglec-1 is upregulated upon DC maturation (26). It is unclear whether this affects antigen presentation, but the enhanced expression of Siglec-1 enhances HIV-1 transmission (27). Since CLR internalization enhances antigen presentation, several strategies have been designed to target these CLRs with antibodies or antigens for vaccination purposes.

### Virus Transmission

Virus internalization can lead to antigen presentation but, strikingly, several CLRs have been shown to protect the virus and promote viral transmission and dissemination (15, 28–30). Virus transmission in the context of DCs can be in *cis*, which depends on productive infection of DCs or in *trans*, where CLRs function as attachment receptors that facilitate capture and transmission without infection of the DC. DC-SIGN is an important CLR on DCs involved in transmission of viruses to susceptible target cells (15), facilitating virus dissemination throughout the host. After DC-SIGN mediated endocytosis of HIV-1, these virions can be kept in multivesicular bodies to enable the release of HIV associated with exosomes. This trans-infection pathway in DCs helps with dissemination of HIV-1 to CD4 + T-cells (31, 32). DC-SIGN also interacts with flaviviruses *via* mannose glycans present on viral envelope glycoproteins and is used by DENV, Hepatitis C virus (HCV) (33), Sindbis virus (34), and the West Nile virus (WNV) for cellular attachment and infection of immature DCs (35, 36). Studies have shown that HCV particles bind to DC-SIGN and are targeted to non-lysosomal compartments in immature DCs, where they are protected from lysosomal degradation and transmit the virus to hepatocytes (33). DC-SIGN functions as an attachment receptor for many different viruses, such as DC-SIGN also mediates Cytomegalovirus (CMV) and Ebola virus (EBOV) enters into DCs and facilitates transmission to susceptible cells in *cis* and *trans* (37, 38). This function is not exclusive for DC-SIGN. Capture of HIV-1 by MR on macrophages also results in protection of the virus and transmission to T-cells (29). Another CLR involved in virus transmission is DCIR. The DCIR expression was detected on DCs, monocytes, macrophages, B-lymphocytes, and granulocytes (23) such as DC-SIGN, DCIR captures HIV-1 and promotes infection in *cis* and *trans* of CD4 + T-cells from immature DCs (28). Inhibiting HIV-1 binding to DCIR on DCs significantly decreases exosomal release of HIV-1 (39), which might be the mechanism for transmission. In contrast, langerin prevents HIV-1 transmission by LCs *via* autophagosomal degradation of HIV-1 (8). When langerin function is impaired, LCs become infected and subsequently transmit HIV-1 to T cells in *cis* (3, 9). In contrast, langerin also functions as an attachment and an entry receptor for influenza A virus and thereby promotes viral dissemination (40). It is unclear how Influenza A virus escapes autophagosomal degradation by langerin. Thus, several viruses have devised strategies to subvert the internalization route of CLRs in order to promote viral dissemination. It is interesting that even though viruses such as HIV-1 escape from degradation in DCs, these DCs still present HIV-1 derived antigens in the context of MHC-II to CD4 + T cells (41). These data suggest that capture by DCs and simultaneous presentation of antigens to CD4 + T cells might enhance the destruction of virus-specific T cells. Moreover, internalization routes of CLRs might be not exclusive for antigen presentation or virus protection, and both routes can occur simultaneously.

### Cross-Presentation

Besides activation of CD4 + T helper (T_H_) subsets, CLRs have also been implied in cross-presentation. *Via* this process, DCs can elicit cytotoxic T cell responses by presenting exogenous antigens *via* MHC class I (42). There are at least two pathways that are generally referred to as “cytosolic” and “vacuolar” (42). Upon endocytosis, antigen processing for MHC class I loading either takes place in endocytic compartments or the cytosol (43–45). Several mechanisms are involved in the transfer of antigens from endosomes into the cytosol and include unfolding of proteins, members of the ER-associated degradation machinery like p97 and the pore-building protein Sec61 as has been reviewed by Schuette and Burgdorf (46). “Cross-priming” describes the subsequent stimulation and expansion of naïve CD8 + T cells to initiate cytotoxic immune responses and memory T cells (47). Even though the exact mechanisms are still under investigation, it is evident that CLRs facilitate uptake that leads to cytosolic exposure and cross-presentation. The BDCA3 + CD141 + DCs excel at cross-presentation and have recently been suggested as the main cross-presenting DC subset in humans, closely resembling mouse CD8 + DCs (48–50). Interestingly, this DC subset expresses CLR CLEC9A (DNGR-1) that efficiently internalizes antigens for cross-presentation (51–53). CLEC9A binds to F-actin (54), which is exposed in necrotic cells and CLEC9A might be involved in cross-presentation of antigens from necrotic cells that have, for example, been infected by viruses. Indeed, murine CLEC9A-deficient DCs are unable to facilitate cross-presentation of Vaccinia virus antigens upon infection (55). CLEC9A might facilitate not only antigen presentation but also activation of CD8 + T cells by presenting signals of tissue damage (55). Moreover, CLEC9A also promotes cross-presentation of dead cell-associated antigens by altering the route of internalization as CLEC9A co-localizes with the phagocytosed necrotic cargo, which diverts the cargo toward the recycling endosomal route for MHC class I presentation (56). Upon infection with the highly immunogenic Herpes simplex virus, cytotoxic CD8 + T cell responses were reduced in mice lacking CLEC9A (56). In humans targeting antigens by CLEC9A antibodies to immature BDCA3 + DCs leads to cross-presentation and induction of antigen-specific CD4 + and CD8 + T cells, whereas DC maturation and cytokine production are not affected (57). Antigens targeted to DCIR on different DC subsets result in efficient cross-priming and cross-presentation (58). DC-SIGN is also able to route viruses or antigens into the cross-presentation pathway as HIV-1 antigens are presented to MHC class I *via* DC-SIGN capture (59, 60). Upon internalization and processing of HIV-1 *via* DC-SIGN, viral exogenous antigens are cross-presented on MHC class I, thereby inducing anti-HIV cytotoxic T cell (CTL) responses (6). Interestingly, the group around Moris et al suggests that the virus in this case is not routed toward lysosomes but is processed by another, proteasome-dependent pathway. Whether langerin is capable of inducing cross-presentation is under debate. Internalization of synthetic long peptides through langerin on LCs enhances cross-presentation (61, 62). Whereas, activated LCs become infected by measles virus (MV) and therefore present newly formed virions *via* MHC class I to MV-specific CD8 + T cells (63). Targeting of MV or MV-infected cells to langerin does not result in cross-presentation, suggesting that langerin routing into BG is not linked to the cross-presentation route (63). MR promotes cross-presentation by routing its cargo into a distinct, low degradative, early endosome subset (7, 64). Importantly, due to poly-ubiquitination of its cytoplasmic tail (65) as well as recruitment of p97 to the endosomal membrane (66), MR might not only internalize antigens but also export the antigens from the endosomal compartment into the cytoplasm (67). The mechanisms of cross-presentation are currently studied extensively in the context of tumor immunology with CLRs as attractive targets (62, 68, 69). However, their impact is also crucial for anti-viral immune responses, and their proven and proposed roles during antigen uptake and presentation depict that CLRs have an important role in connecting these processes.

## Shaping Antiviral Innate Immune Responses by CLRs

PRR signaling in DCs is vital to the induction of innate and adaptive immune responses to viruses. Type I IFN responses are paramount in limiting viral replication and therefore form a strong innate immune defense mechanism against invading viruses (70–73). Moreover, type I IFN responses also modulate adaptive immunity thereby further tailoring immunity to the pathogen. Different CLRs possess the capacity to activate various innate signaling pathways that give rise to specific types of cellular immune responses (Figure 2) (74). However, viruses contain the capacity to alter CLR-induced signaling, thereby inhibiting induction of type I IFN responses (75).

### Type I IFN Responses in DCs

Sensing of viral structures *via* a variety of PRRs induces an antiviral program to help viral infections (76–78). This innate antiviral program consists predominantly of various IFNα subtypes and IFNβ, which has been reviewed extensively elsewhere (79). IFNα and IFNβ are both produced by DCs, but IFNα is predominantly secreted by pDCs (80–82). Membrane-bound TLRs trigger signaling cascades leading to phosphorylation of interferon-regulatory factor 3 (IRF3) and IRF7 activate transcription of *IFNA* and *IFNB* genes (82, 83). IRF3 and IRF7 are crucial in inducing type I IFN, albeit in a different manner. IRF3 is indispensable for the first production of type I IFN and predominantly activates IFNβ signaling, which then initiates transcription of IRF7 thereby strongly inducing IFNα. IRF7 in contrast to IRF3 is not constitutively expressed by DCs, and is upregulated by IFN signaling. Therefore, IRF7-induced IFNα is under control of IRF3 activation and thus IFNβ production (4, 78, 82, 83). Therefore, IFNβ comprises the very first line of antiviral defense as it forms the first wave of type I IFN. Secreted IFNα/β proteins bind the heterodimeric transmembrane IFN receptors (IFNARs) on the cell surface (78). Upon receptor ligation, intracellular signaling leads to induction of *IFNA/B* gene transcription and transcription of interferon stimulated genes (ISGs) Depending on the cell type, IFN dosage and timing of the first wave of IFN exposure, 50–1000 ISG can be identified among 200–500 different cell types (84). ISGs form an important antiviral type I IFN component, since they can interfere with viral replication steps or serve as viral restriction factors (70–73). Thus, upon transcription of *IFNA/B* genes a self-enhancing antiviral program is induced that can strongly inhibit viral replication. In addition, the production of type I IFN is essential to adaptive immunity as these cytokines control proliferation, differentiation, activation and maturation of monocytes, DCs, and macrophages (85). Therefore, type I IFN plays a crucial role in the induction of antiviral immunity.

### Shaping of Innate Immune Responses by CLRs

C-type lectin receptors are important in inducing and modulating immunity (Figure 2). Some CLRs contain immunoreceptor tyrosine-based activation motifs (ITAMs), hemi-ITAMs, or immunoreceptor tyrosine-based inhibitory motifs (ITIMs), whereas some do not contain any obvious signaling motifs (75). Dectin-2, DCAR, and MDL-1 belong to the ITAM-containing CLRs, whereas DCIR contains an ITIM. Dectin-1 belongs to the hemi-ITAM group, and other CLRs such as DC-SIGN, MR, and DEC-205 do not contain any known ITAMs or ITIMs (75). DCIR is expressed by DCs and macrophages, and contains an ITIM that mediates inhibitory signals by recruiting phosphatases SH2-domain-containing protein tyrosine phosphatase 1 (SHP1) or SHP2 after receptor ligation (86). Endocytosis of DCIR on DCs does not affect TLR4 and TLR8-dependent DC maturation (87). However, DCIR inhibits TLR8-dependent IL-12 and TNFα production, whereas TLR2, TLR3, and TLR4-induced cytokine levels are unaffected (87). DCIR is also expressed on macrophages, and its activation inhibits CpG-ODN-induced expression of pro-inflammatory cytokines IL-1β and IL-6 (88). Although DCIR inhibits IFNα (19), it also serves an opposing role by sustaining type I IFN responses (89) as murine DCs deficient in DCIR have decreased STAT1 phosphorylation upon *Mycobacterium tuberculosis* infection, indicating that DCIR sustains STAT1 and therefore type I IFN responses (89). Notably, DCIR expression inhibits IL12p70 production and DC-dependent T_H_1 skewing (89). Thus, DCIR depending on the DC subset can inhibit or sustain type I IFN responses depending on the pathogen and cell-type. Whether DCIR is able to sustain type I IFN responses in humans and against viruses remains elusive. DCIR contributes to DC capture and dissemination of HIV-1, whereas murine DCIR has shown to be involved in the internalization of Chikungunya virus (CHIKV) (28, 90). Whether DCIR, in this context, functions as a PRR and is able to sustain type I IFN responses remains elusive. HIV-1 induces DCIR expression on T cells, which increases viral entry and replication (91) whereas DCIR serves a protective role in CHIKV infection (90). Thus, depending on the virus and on the origin of the host cell, DCIR appears to either protect against or enhance viral infection of the host cell. BDCA-2 (CLEC4C, CD303) is expressed by pDCs and therefore widely used to identify pDCs (92). BDCA-2 inhibits type I IFN responses in pDCs in response to CpG oligonucleotides, Influenza virus, or DNA-autoantibody complexes (93, 94). Similar to Dectin-2 and DCAR, BDCA-2 contains the capacity to engage with the transmembrane adaptor FcεRIγ (95) and induce ITAM-dependent signaling (96). BDCA-2 crosslinking with antibodies on pDCs inhibits both type I IFN and pro-inflammatory cytokine responses after TLR7 and TLR9 triggering (96). Interestingly, BDCA-2 on pDCs interacts with HCV glycoprotein E2, which similarly blocks type I IFN responses (97). MR binding to DENV on macrophages induces pro-inflammatory cytokines TNFα and IL-1β, causing decreased endothelial integrity and leading to more severe disease symptoms (11, 98). As MR expression is increased during DENV infection *via* IL-4, this suggests that MR-induced cytokines might be important in sustained high cytokine responses (99, 100). Recent findings have shown that when macrophages are stimulated with vitamin D3, IL-4-induced MR upregulation is attenuated and thus leads to decreased ligation of viral particles to macrophages (101). MDL-1 (CLEC-5A) also interacts with DENV and induces Syk-dependent pro-inflammatory IL-1β and IL-18 responses upon DENV-induced inflammation (102). Ligation of MDL-1 activates the NLRP3 inflammasome and pyroptosis (102). In macrophages, MDL-1 is exploited by Japanese encephalitis virus, which uses this inflammatory axis to induce virus-dependent inflammation of host cells (103). Moreover, MDL-1 binding to Influenza A virus (104, 105) contributes to Influenza A virus pathogenicity through induction of pro-inflammatory responses in a murine model (105). HIV-1 binding to DC-SIGN induces signaling by DC-SIGN that affects TLR-mediated signaling. Endosomal degradation of HIV-1 triggers TLR8-dependent NF-κB and initiates transcription of integrated HIV-1 (106, 107). Notably, DC-SIGN signaling by HIV-1 induces phosphorylation of NF-κB allowing propagation of transcription and production of viral proteins (108). Thus, DC-SIGN-dependent endocytosis and signaling is exploited by HIV-1. Furthermore, a recent study showed that HIV-1 targets DC-SIGN on primary DC subsets to suppress type I IFN responses induced by the HIV-1 sensor DDX3 (109). Similarly, MV exploits DC-SIGN signaling to suppress type I IFN responses as DC-SIGN signaling blocks phosphatases that are crucial in activation of MV-sensors RIG-I and MDA5 (110). Thus, both HIV-1 and MV exploit DC-SIGN signaling to inhibit antiviral type I IFN responses.

## CLRs in T Helper Cell Polarization

Efficient pathogen-specific T cell responses require differentiation of CD4 + T cells into various T_H_ cell subsets (Figure 1). Distinct T_H_ cell subsets each have specialized roles in the defense against invading pathogens. A T_H_1 response is directed against intracellular pathogens, whereas T_H_2 cells produce IL-4, IL-5, and IL-13 to combat extracellular pathogens (111). Follicular T_H_ cells (T_FH_) are crucial for efficient B cell responses by the formation of germinal centers in the lymph node. In these germinal centers, T_FH_ stimulate B cell proliferation and isotype class-switching *via* the production of IL-21 (112, 113). CLRs crosstalk with other PRRs to induce specific cytokine expression profiles thereby directing T_H_ cell polarization (114). DC-SIGN distinguishes between mannose- and fucose-containing antigens (14, 115, 116). Notably, differential recognition of mannose and fucose structures by DC-SIGN results in the induction of disparate intracellular pathways that are controlled by the composition of the signalosome bound to DC-SIGN (112, 117, 118). DC-SIGN is continuously bound by adaptor protein LSP1 in combination with a signalosome complex KSR1, CNK, and kinase Raf-1 (Figure 2) (117). Activation of DC-SIGN by mannose-containing pathogens such as HIV-1 or MV leads to activation of Raf-1. Raf-1 triggers a signaling pathway that induces a specific phosphorylation of NF-κB, thereby enhancing the transcription of pro-inflammatory cytokines IL-6 and IL-12 (Figure 2) (74, 119). High mannose structures are prevalent on the surface of many viruses including HIV-1, EBOV, HCV, DENV, CMV, and SARS coronavirus (SARS-CoV) (74). Therefore, the interaction of these viruses with DC-SIGN might enhance the induction of T_H_1 responses (120). In contrast, activation of DC-SIGN by fucose-expressing pathogens dissociates the Raf-1 signalosome from LSP1 and recruits IκB kinase subunit-ε (IKKε) and the de-ubiquitinase (CYLD) (Figure 2) (112, 118). CYLD activation results in accumulation of ubiquitinated Bcl3 in the nucleus, which forms p50–p50–Bcl3 complexes that inhibit IL-12 production and enhances T_H_2-associated cytokines IL-10, and CCL17 and CCL22 (Figure 2) (118). Indeed fucose-containing pathogens induce T_H_2 polarization *via* DC-SIGN activation (118). Furthermore, fucose-induced signaling by DC-SIGN also modulates IFNR signaling, which is paramount to the induction of IL-27 and T_FH_ cells (112). Mostly parasites express fucose, suggesting that DC-SIGN is important in the defense against parasites that require T_H_1 and T_FH_ responses. Interestingly, a few viruses, such as DENV, expose fucose-structures on their surface. However, it remains unclear whether these viral fucose–DC-SIGN interactions induce T_H_2 responses. It might be possible that other CLRs also induce T_H_2 fucose-dependent differentiation. CLRs such as langerin and MR also interact with LSP1, suggesting that the signaling properties of these receptors are similar to DC-SIGN. Besides DC-SIGN, other receptors are also involved in T cell polarization. MR is known to inhibit the production of TLR4-induced IL-12 secretion in DCs (121). Among DCs, DCIR and myeloid inhibitory C-type lectin-like receptor (MICL) both down-regulate TLR-induced IL-12 secretion *via* activation of intracellular ITIM motifs (87, 122). In addition, BDCA2 downregulates TLR-mediated IL-6 production by preventing recruitment of MyD88 to the intracellular domains of TLRs in pDCs (93). Therefore, activation of one or more of these CLRs might skew toward a T_H_2 balance. However, most of these studies used bacterial ligand such as LPS to investigate cytokine response upon CLR stimulation. Additional research is needed to investigate whether these receptors still exhibit these signaling modulating functions upon viral infections.

## Crosstalk Between CLRs and Complement Receptors (CRs)

**Figure 3:**
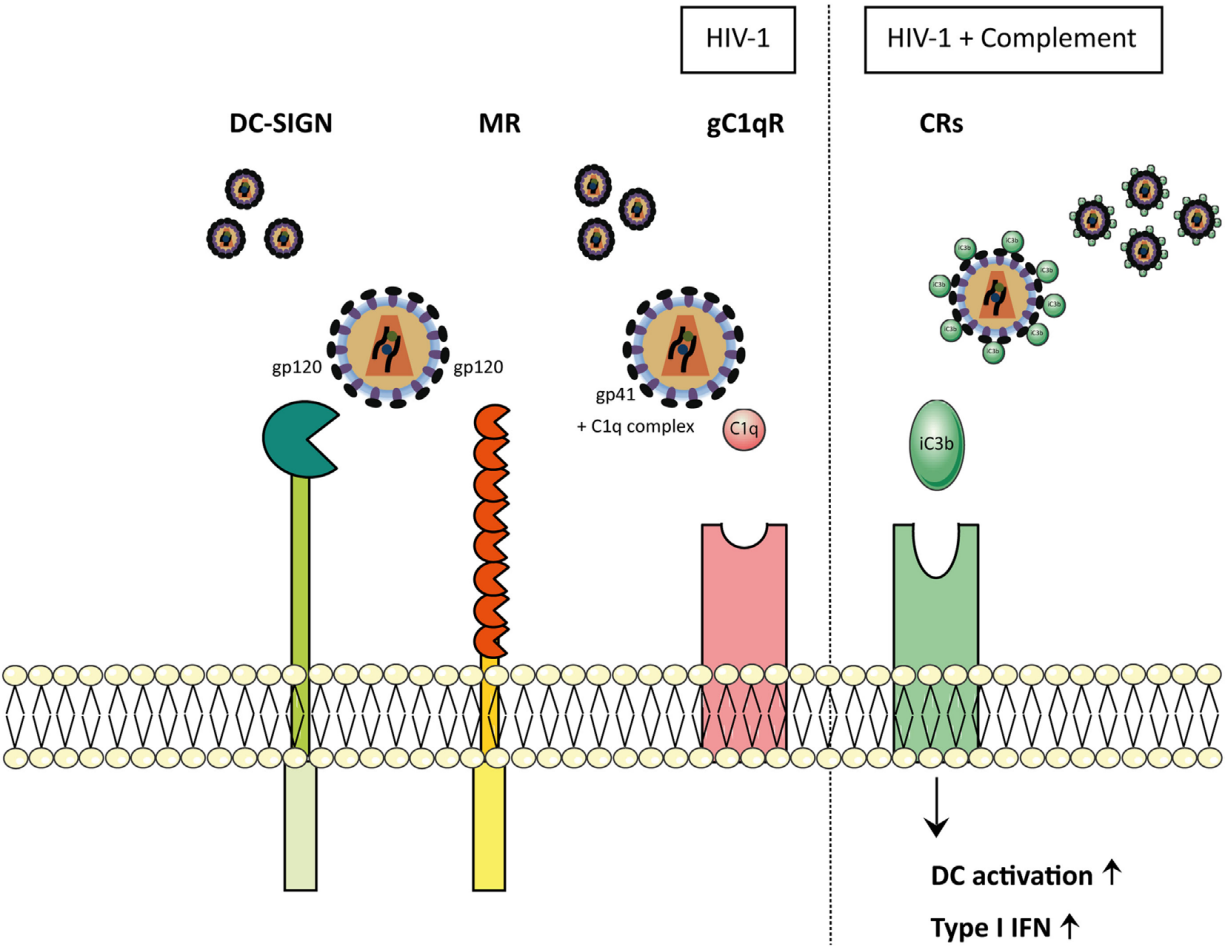
HIV-1 particles opsonize-dependent recognition on myeloid cells. In physiological conditions, when HIV-1 enters the body, it is either non-opsonized (HIV) or complement-opsonized (HIV-C). Depending on the opsonization pattern of the virions, interactions with the receptors differ. Non-opsonized virus (HIV-1) interacts with CLR receptors, such as DC-SIGN and mannose receptor (MR) *via* the glycoprotein gp120. Furthermore, non-opsonized virus (HIV-1) is able to establish an interaction between gp41 and C1q complex, allowing the final binding to gC1qR. MR and C1qR cause the activation of the complement system *via* the lectin pathway and the classical pathway, respectively, inducing complement-mediated opsonization of the virions. Once HIV is coated by C3b fragments, it is able to bind to the complement receptors (CRs). Dendritic cells (DCs) exposed to complement-opsonized HIV-1 showed increased activation as well as up-regulation of type I IFNs.

C-type lectin receptors are also involved in complement-dependent clearance of pathogens as well as induction of immunity. The complement system can be activated through three distinct pathways, named the classical, the lectin, and the alternative pathway (123). The three different cascades are responsible in activating complement factor C3b that deposits on diverse surface formations of pathogens and apoptotic cells inducing opsonization, inflammation, phagocytosis, elimination of the pathogen, and lastly, the induction of adaptive immune responses. Interestingly, the soluble CLR MBL is involved in the lectin pathway and recognizes carbohydrates such as mannose, glucose, l-fucose, *N*-acetyl-mannosamine (ManNAc), and *N*-acetyl-glucosamine (GlcNAc) on a wide range of pathogens (124). The recognition of carbohydrates is mediated *via* the CRDs and oligomerization of MBL enables high avidity binding to repetitive carbohydrate ligands. Once bound, MBL has the capacity to modify the efficiency of uptake by the expression of other phagocytic receptors. MBL further activates complement, due to its association with Mannose-binding lectin-Associated Serine Proteases, or act directly as an opsonin (125, 126), which results in deposition of complement on the pathogen surface that leads to uptake *via* CRs (126, 127). MBL has been found to bind directly to virions from a number of different virus families, including HIV, SARS-CoV, EBOV, DENV, and WNV. For instance, MBL binds directly to HIV-infected cells (128) and HIV-1 particles that lack gp120 do not bind MBL, supporting the idea that gp120 and gp41 directly bind to C1q and guide the interaction between the whole virus and MBL (129, 130). Despite these findings, the role of MBL in HIV pathogenesis is still unclear. Moreover, complement also affects CLR function. When entering the host, complement is spontaneously deposited on the surface of HIV-1 since gp120 and gp41 contain a C1q binding site (131). Over the past years, a lot of studies have supported the idea that HIV-1 envelope glycoprotein gp41 functions as a viral ligand for gC1qR (132–135), the receptor for the globular heads of C1q complex, which modulates a plethora of immunological functions, such as infection and inflammation. The HIV-1 transmembrane glycoprotein gp41 has been shown to interact with the C1q complex (136, 137), causing the activation of the classical pathway of the complement system (Figure 3). Furthermore, different studies have shown that MBL interacts with HIV-1. Expression of viral proteins, gp120 and gp41, seems to be crucial for the HIV-1-MBL interaction. However, the binding is presumably to the *N*-linked glycans on the gp120 (Figure 3), due to the fact that it is highly glycosylated and in contrast there are only few potential *N*-linked carbohydrate sites on gp41 (138, 139). MBL interaction with the envelope glycoprotein of different HIV-1 strains mediate several downstream antiviral effects, in particular the complement activation, independent of C1q and antibodies, inducing the lectin pathway and enhancing the opsonization and viral elimination (128, 140). Complement is important early in infection, when HIV-1 specific antibodies are still absent. Complement is active at the mucosal site and in the seminal fluid, which suggests that the virus is opsonized with C3b prior to transmission (141). Opsonization reduces the accessibility of the viral envelope protein gp120, and interferes with the CLR interaction of the virus with DCs (142). In addition, complement opsonization of HIV-1 causes a significantly higher productive infection of DCs, which is caused by binding to the CR3, whereas non-opsonized HIV-1 is bound *via* gp120 to DC-SIGN (143) (Figure 3). However, complement is a two-edged sword and besides its clearance and neutralization activity, it also enhances viral spread and maintenance. Thus, complement-coated HIV-1 accumulates in different parts of the host and is able to bind CR-expressing immune cells, for example DCs, macrophages, NK cells, B cells, or even follicular DCs, thereby enhancing infectivity and dissemination (130, 144). In addition, novel complement activation pathways have been identified such as interaction of C1q with CLR SIGN-R1 expressed on macrophages (145), and cells with a DC-like phenotype (146). It has been widely accepted that infection of DCs by HIV-1 and DC-mediated transmission of the virus to T cells is mediated by CLRs (143), but this is not the case for opsonized virus. The productive infection caused by HIV-C is associated with an activation of DC responses characterized by up-regulation of maturation (CD83, CCR7), co-stimulatory function (CD40 and CD86), together with HLA-DR and HLA-ABC. Furthermore, innate type I IFN responses are enhanced that are also involved in T cell activation (CXCL9, CXCL10, CXCL11), suggesting that there is also an improved antiviral response (147) (Figure 3).

## Concluding Remarks

Some CLRs are important in shaping innate and adaptive immunity to different viruses. Many viruses interact with CLRs and the elicited immune responses are induced by triggering of CLRs in combination with PRRs. Although the intracellular signaling pathways have not been clearly defined for most CLRs, it is becoming evident that CLRs are very efficient in modulating signaling by other PRRs. This seems to be a recurring feature that is important in tailoring adaptive immunity to the pathogens. However, certain viruses have subverted these CLRs to inhibit antiviral immunity. In particular, several CLRs have been shown to inhibit type I IFN responses, which is crucial for a strong effective antiviral immune response. Moreover, although CLRs are currently main players in immunotherapy strategies to enhance antigen presentation to tumor antigens, the intracellular routing of CLRs is subverted by many viruses for viral transmission. These pro-virus functions might be natural functions of the CLRs and it is important to further understand these functions and why CLRs require these in inducing immunity. It is likely that these CLRs recognize other pathogens and that the subsequent immune responses require limiting amounts of type I IFN responses or pro-inflammatory cytokines. The elucidation of the mechanisms behind these manipulations is crucial to target CLRs in order to combat viral infections or to prevent viral invasion and is also important to understand inflammatory as well as auto-immune diseases.

## Author Contributions

MB, JE, LH, NH, BN, MS: wrote sections of the manuscript, contributed equally. TG: wrote sections of the manuscript, supervision and final responsibility. All authors contributed to manuscript revision, read and approved the submitted version.

## Conflict of Interest Statement

The authors declare that the research was conducted in the absence of any commercial or financial relationships that could be construed as a potential conflict of interest.
